# Ethnicity and spatiotemporal parameters of bilateral and unilateral transtibial amputees in a 100-m sprint

**DOI:** 10.1186/s40064-016-1983-1

**Published:** 2016-03-17

**Authors:** Hiroaki Hobara, Satoru Hashizume, Yoshiyuki Kobayashi, Yuko Usami, Masaaki Mochimaru

**Affiliations:** Human Informatics Research Institute, National Institute of Advanced Industrial Science and Technology (AIST), Waterfront 3F, 2-3-26, Aomi, Koto-ku, Tokyo 135-0064 Japan

**Keywords:** Running-specific prostheses, Prosthetic sprinting, Step frequency, Step length

## Abstract

Similar to able-bodied sprinters, most of the medals for the 100-m sprint in past Paralympic Games and IPC Athletics World Championships were dominated by West African (WA) and Caucasian (CC) amputee sprinters, not Asian (AS) sprinters. Although these results indicate differences in sprint performance due to ethnicity, little is known about the ethnicity and spatiotemporal parameters of the 100-m sprint for amputee sprinters. The purpose of this study was to investigate the differences in the spatiotemporal parameters of WA, CC and AS sprinters with bilateral and unilateral transtibial amputations during a 100-m sprint. We analyzed 6 WA, 28 CC, and 10 AS amputee sprinters from publicly available Internet broadcasts. For each sprinter’s run, the average speed, average step length, and step frequency were calculated by using the number of steps in conjunction with the official race time. No significant differences were found in the spatiotemporal parameters of the 100-m sprint for the WA and CC groups. On the other hand, the average speed of the AS group was significantly lower because of its shorter step length during the 100-m sprint. The results suggest that WA and CC sprinters would perform similarly during a 100-m sprint, but AS sprinters would not.

**Electronic supplementary material:**

The online version of this article (doi:10.1186/s40064-016-1983-1) contains supplementary material, which is available to authorized users.

## Background

The development of running-specific prostheses (RSPs) has allowed runners with initial lower extremity amputations to compete at levels never before achieved (Hobara et al. 2015a). Theoretically, the average speed during a 100-m sprint is the product of the average step frequency and average step length. Since spatiotemporal parameters change by sprint training sessions (Bezodis et al. 2008), an increased understanding of spatiotemporal parameters during a 100-m sprint in amputee sprinters will provide us with a basis for better evaluating changes in sprint performance which accompany training regimes and would be expected to aid in the development of more effective training methods in this population (Bezodis et al. 2008; Salo et al. 2011).

According to the 2015 International Paralympic Committee (IPC) Athletics Official World Rankings (T43 and T44 classes), the fastest times by West African (WA) sprinters (10.61 s) and Caucasian (CC) sprinters (10.71 s) have a gap of only 0.10 s (as of December 31). On the other hand, the Asian (AS) record is 12.08 s, which is much less than the WA and CC records. Similar to able-bodied sprinters, most of the medals for the 100-m sprint in past Paralympic Games and IPC Athletics World Championships were dominated by WA and CC amputee sprinters, not AS sprinters. These results indicate differences in sprint performance due to ethnicity may exist in amputee sprinters.

Despite the fact that several studies demonstrated ethnicity-related architectural and functional differences of the musculoskeletal system in able-bodied athletes (Fukashiro et al. 2002; Kunimasa et al. 2014; Rahmani et al. 2004), little is known about the ethnicity and athletic performance in athletes with lower extremity amputations. Therefore, the purpose of this study was to investigate the differences in spatiotemporal parameters of AS, CC, and WA sprinters with bilateral and unilateral transtibial amputations during a 100-m sprint. We hypothesized that the WA and CC groups would perform similarly in the 100-m sprint, but the AS group would not.

## Methods

In total, we analyzed 44 sprinters with bilateral and unilateral transtibial amputations from publicly available Internet broadcasts. Based on the classification system created by the IPC, we included the Men’s T43 class (i.e., double below-knee amputees and other athletes with impairments comparable to a double below-knee amputation) and Men’s T44 class (i.e., any athlete with lower limb impairments that meet the minimum disability criteria for lower limb deficiency, impaired lower limb passive range of motion, impaired lower limb muscle power, or leg length difference). These races included several Paralympics, the IPC Athletics World Championships, and other national- and international-level competitions from 1996 to 2015 (Table 1). We only included the fastest time for each individual into the dataset of each respective group. Individual races were excluded from the analysis if the athlete did not complete the race or the athlete’s body was not visible throughout the entire race. T43/44 sprinters who did not use RSPs were also excluded. Prior to initiation of this study, institutional review board approval was obtained. All ethical standards were also maintained during the conducting of this research.

**Table 1:** Summary of the competitions analyzed

Year	Competitions	Number of subjects		
		WA	CC	AS
2015	IPC athletics	1	3	
2015	Mano a mano challenge		1	
2015	SEIKO super athletics		1	
2015	Parapan 2015		1	
2015	IPC Grand Prix London		1	
2015	IPC Grand Prix Dubai		1	
2015	Shizuoka International			2
2014	Japan Nationals			1
2014	Great City Games Manchester	1		
2013	International Wheelchair and Amputee Sports Games		2	
2013	Sainsbury’s Anniversary Games		2	
2013	IPC Athletics		5	
2013	Shizuoka International	2		1
2012	Mt. Sac Relays		2	
2012	London Disability Athletics Challenge			1
2012	London Paralympic	1	1	
2012	IPC European Championship		1	
2011	International Wheelchair and Amputee Sports Games		1	
2011	Oita Athletics			1
2011	JPN National			1
2010	Asia Paralympic			1
2011	Japan Paralympic			1
2011	IPC Athletics		2	
2009	Manchester BT Paralympic World Cup	1	1	
2008	Beijing Paralympic		2	
1996	Atlanta Paralympic		1	
	Total	6	28	10

In the present study, we separated the whole population into three groups based on ethnicity: 6 WA, 28 CC, and 10 AS sprinters. Number of sprinters who satisfied A- and B-Qualification standards in T43/44 (12.20 s and 12.50 s, respectively) were obtained (WA; AQS 6: BQS 0, CC; AQS 23: BQS 1, AS; AQS 2: BQS 3). In previous studies (Hobara et al. 2015b, 2016a, b), we determined the average speed in the 100-m sprint (*S*_100_) of each individual by dividing the race distance (100 m) by the official race time (*t*_*race*_) from each competition’s official website:

1 $$S_{100} = \, 100/t_{race} .$$

We calculated the average step frequency (*f*_step_) as

2 $$f_{step} = N_{step} /t_{race} ,$$

where *N*_step_ was the number of steps, which was manually counted by the authors. If we could not count the number of steps, we excluded the data from our analyses. The last step before the finish line was considered to be the last step. If an athlete’s foot was located on the finish line, we considered it as a step. Further, we calculated *L*_step_ by

3 $$L_{step} = S_{100} /f_{step} .$$

Before we interpreted the results, we performed the Shapiro-Wilks and Levene tests to ensure that the assumptions of normality and homogeneity of variance were met. The tests revealed that our groups were homogenous. One-way analysis of variance (ANOVA) was performed to compare *S*_100_, *f*_step_, and *L*_step_ (dependent variables) of the WA, CC and AS sprinters (independent variables). We also calculated effect sizes (ES: 0.4, 1.15 and 2.70 for small, medium, and strong, respectively) for each ANOVA (Ferguson 2009). Bonferroni post hoc multiple comparison tests were performed if a significant main effect was observed. Further, Pearson’s correlation coefficients were used to examine the relationship between *S*_100_, *f*_step_ and *L*_step_ in three groups. Statistical significance was set at *P* < 0.05. These statistical analyses were executed by using SPSS version 19 (IBM SPSS Statistics Version 19, SPSS Inc., Chicago, IL).

## Results

**Fig. 1:**
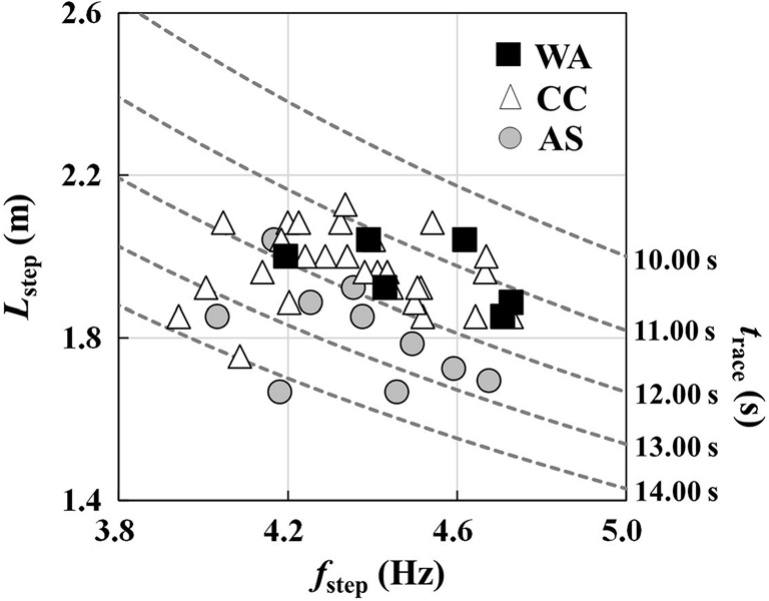
Relationships between *f*_step_ and *L*_step_ for the three groups. The *gray circles*, *unfilled triangles*, and *black squares* indicate the data for West African (WA), Caucasian (CC), and Asian (AS) sprinters, respectively. The *dotted lines* denote the predicted times calculated with *f*_step_ and *L*_step_

**Fig. 2:**
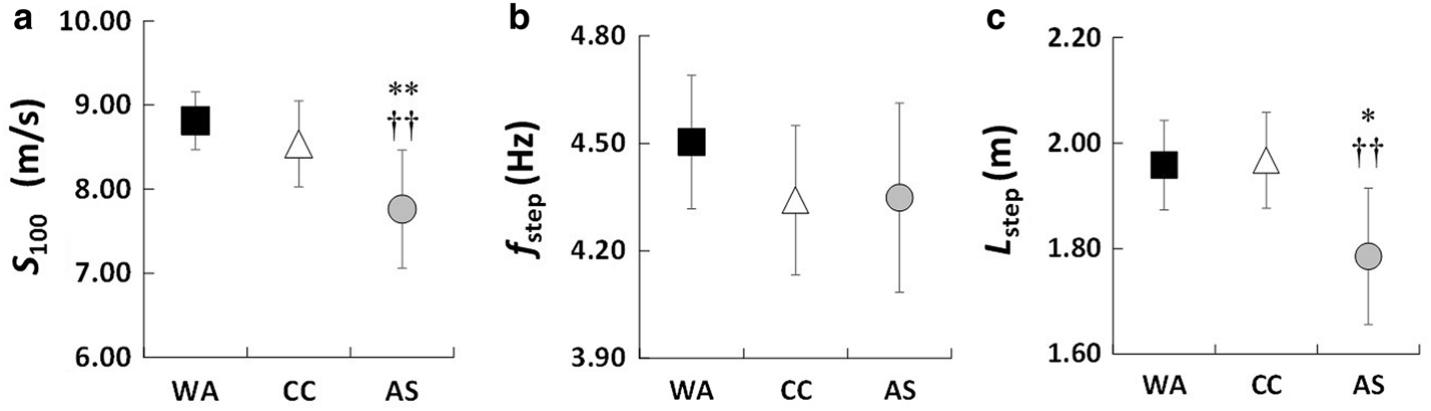
Comparison of **a** averaged speed (*S*_100_), **b** step frequency (*f*_step_), and **c** step length (*L*_step_) of West African (WA), Caucasian (CC) and Asian (AS) sprinters. The *daggers* (††) indicate significant differences from the CC group at *P* < 0.01. The *asterisks* (*) indicate significant differences from the WA group at *P* < 0.05

Figure 1 shows the *f*_step_–*L*_step_ plot for all of the individuals in the three groups. The dotted lines indicate the times predicted by using *f*_step_ and *L*_step_ together. As shown in Fig. 2a, *S*_100_ exhibited a significant main effect on the groups (*F*_(2, 41)_ = 8.90, *P* < 0.01, ES = 0.19; small). Although there was no significant difference in *S*_100_ between the WA and CC groups, *S*_100_ was significantly lower for the AS group (*P* < 0.01). However, there were no significant main effect between the groups for *f*_step_ (*F*_(2,41)_ = 1.46, *P* = 0.24; ES = 0.01; small, Fig. 2b). The statistical analysis also revealed the presence of a significant main effect on the groups by *L*_step_ (*F*_(2, 41)_ = 9.63, *P* < 0.01, ES = 0.22; small, Fig. 2c). There was no significant differences in *L*_step_ between the WA and CC groups, while *L*_step_ was significantly shorter for the AS group. Descriptive data of per each dependent variable were also calculated (see Additional file 1).

As shown in Table 2, both *f*_step_ (*r* = 0.545) and *L*_step_ (*r* = 0.385) were not significantly correlated with *S*_100_ in WA. On the other hands, both *f*_step_ (*r* = 0.680, *P* < 0.01) and *L*_step_ (*r* = 0.660, *P* < 0.01) were significantly correlated with *S*_100_ in CC. Although there was no significant correlations between *f*_step_ and *S*_100_ in AS (*r* = 0.191), but *L*_step_ were significantly correlated with *S*_100_ (*r* = 0.749, *P* < 0.05). We also found a negative linear relationship between *f*_step_ and *L*_step_ in all groups, but it did not reach significance (WA; *r* = −0.563: CC; *r* = −0.101: AS; *r* = −0.507).

**Table 2:** Pearson’s correlation coefficient in three groups

	WA	CC	AS
*S*_100_—*f*_step_	0.545	0.680**	0.191
*S*_100_—*L*_step_	0.385	0.660**	0.749*
*f*_step_—*L*_step_	−0.563	−0.101	−0.507

*^,^ ** Significance at *P* < 0.05 and 0.01, respectively

## Discussion

Our results showed no significant differences in the spatiotemporal parameters of the WA and CC groups running a 100-m sprint. On the other hand, *S*_100_ was significantly lower for the AS group because of their shorter *L*_step_ for the 100-m sprint (Fig. 2a, c). The results agree with our initial hypothesis that the WA and CC groups would perform similarly in the 100-m sprint, but the AS group would not.

A previous study found differences in the muscle and tendon viscoelastic property indices of the triceps surae between African and Caucasian athletes (Fukashiro et al. 2002). In addition, past findings (Abe et al. 1999; Rahmani et al. 2004) have shown that Senegalese (West African origin) have longer legs and lower moment of inertia of limb than Italians (representative of South and West Europe populations). These results suggest that these ethnic differences may influence the running performance of able-bodied athletes. On the other hand, our current data are based on amputee sprinters using RSPs. RSPs have lower mass, a smaller moment of inertia, and higher elasticity than intact human shank–foot segments (Baum et al. 2013; Brüggemann et al. 2008). Such mechanical characteristics may offset the inherent musculoskeletal bias between CC and WA sprinters, which would lead to the similar spatiotemporal parameters for both groups.

The differences in spatiotemporal parameters between the AS group and two other groups may be explained by the differences in muscle and tendon architectures. *L*_step_ during sprinting partly depends on the vertical and horizontal ground reaction forces (GRFs) and impulses (Hay 1994). A previous study demonstrated that African runners have longer lower extremities and Achilles tendons than Japanese runners (Kunimasa et al. 2014). Furthermore, Caucasian patients seem to have longer hamstring tendons than Chinese patients (Chiang et al. 2012). In addition, it has been shown that African and European generally has longer lower extremities than Asian (Pheasant 1986). Therefore, differences in *L*_step_ between the AS group and two other groups may be due to ethnicity-related architectural and functional differences of the musculoskeletal system in lower extremities that cannot be offset by using RSPs.

As shown in Table 2, we also found that there were no significant correlation between *f*_step_, *L*_step_ and *S*_100_ in WA. Further, although both *f*_step_ and *L*_step_ were significantly correlated with *S*_100_ in CC, only *L*_step_ was significantly correlated with *S*_100_ in AS (Table 2). These results indicate that determinants of sprint performance are not the same among different ethnicities in sprinters using running-specific prostheses.

There are some limitations in this study. First, we calculated average step length using the number of steps taken and the time as data. However, all the steps would not be of the same length. For example, a lot of short steps may be taken in the initial acceleration phase from the start. Thus, current data should be recognized as ‘averaged’ step rate and length across the distance. Second, miscounting steps based on videos from open internet sources might influence subsequent calculation of step frequency and step length. Although we excluded the data from our analyses if we could not count all the steps, we are aware of the possibility of miscounting when the camera view switched in order to follow the athletes. Further, although we calculated spatiotemporal parameters using official race time and the number of steps taken, the athlete would not necessarily complete a step exactly at 100 m. Indeed, Salo et al. (2011) subtracted a distance of 0.55 m and a time of 0.52 s from the calculations of averaged step length and step frequency based on their pilot test. Therefore, caution needs to be taken regarding the interpretation and generalization of these findings. Thirdly, although most of T43/44 sprinters after 1996 generally use Flex-Foot Cheetah^®^ (Össur), Cheetah^®^ Xtreme™ (Össur) or 1E90 Sprinter (Ottobock), we did not determine individual’s RSPs, which might influence spatiotemporal parameters during sprinting. Thus, caution needs to be taken regarding the interpretation and generalization of these findings. Finally, the difference in number of subjects among three groups is large, which may affect the significant level, such as significance in the Pearson’s correlation coefficient. Therefore, further research is needed to clarify the relationship between ethnicity and spatiotemporal parameters during a 100-m sprint in amputee sprinters.

Theoretically, average forward velocity in a 100-m sprint is the product of average *f*_step_ and average *L*_step_. Although both parameters are inversely correlated, an increase in one factor will result in an improvement in sprint velocity, as long as the other factor does not undergo a proportionately similar or larger decrease. Therefore, an increased understanding of spatiotemporal parameters during 100-m sprint will provide coaches and practitioners with a basis for better evaluation of the changes in sprint performance and aids in the development of more effective training methods for amputee sprinters. Furthermore, identifying factors affecting these spatiotemporal parameters of 100-m sprints in amputee sprinters could be beneficial to optimal selection and newly-development of running-specific prostheses.

## Conclusion

In this study, we investigated the differences in spatiotemporal parameters of WA, CC, and AS sprinters with bilateral and unilateral transtibial amputations running a 100-m sprint. The results indicate that the WA and CC groups performed similarly, but the AS group did not.

## Additional file

10.1186/s40064-016-1983-1 Descriptive data of per each dependent variable.
