# Supplementary material for: Ethnicity and spatiotemporal parameters of bilateral and unilateral transtibial amputees in a 100-m sprint
Source: Springerplus. 2016 Mar 17;5:343. doi: 10.1186/s40064-016-1983-1 (PMC4794476; doi:10.1186/s40064-016-1983-1)
Supplement: Supplementary file 1 — 10.1186/s40064-016-1983-1 Descriptive data of per each dependent variable. [file 40064_2016_1983_MOESM1_ESM.docx]

**Supplementary material 1.** Descriptive data of per each dependent variable

|  |  | **WA** | **CC** | **AS** |  |
| --- | --- | --- | --- | --- | --- |
|  | *S*_100_ |  |  |  |  |
|  | Mean | 8.82 | 8.56 | 7.87 |  |
|  | SD | 0.37 | 0.53 | 0.46 |  |
|  | 95%CI | 8.43, 9.21 | 8.35, 8.77 | 7.54, 8.21 |  |
|  |  |  |  |  |  |
|  | *f*_step_ |  |  |  |  |
|  | Mean | 4.51 | 4.35 | 4.36 |  |
|  | SD | 0.21 | 0.21 | 0.20 |  |
|  | 95%CI | 4.29, 4.73 | 4.27, 4.44 | 4.21, 4.50 |  |
|  |  |  |  |  |  |
|  | *L*_step_ |  |  |  |  |
|  | Mean | 1.96 | 1.97 | 1.81 |  |
|  | SD | 0.08 | 0.09 | 0.23 |  |
|  | 95%CI | 1.87, 2.04 | 1.93, 2.00 | 1.72, 1.90 |  |
|  |  |  |  |  |  |
